# Evidence of the Physical Interaction between Rpl22 and the Transposable Element *Doc5*, a Heterochromatic Transposon of *Drosophila melanogaster*

**DOI:** 10.3390/genes12121997

**Published:** 2021-12-16

**Authors:** Maria Francesca Berloco, Crescenzio Francesco Minervini, Roberta Moschetti, Antonio Palazzo, Luigi Viggiano, René Massimiliano Marsano

**Affiliations:** 1 Department of Biology, University of Bari “Aldo Moro”, 70126 Bari, Italy; mariafrancesca.berloco@uniba.it (M.F.B.); roberta.moschetti@uniba.it (R.M.); antonio.palazzo@uniba.it (A.P.); 2 Department of Emergency and Organ Transplantation (D.E.T.O.), Hematology and Stem Cell Transplantation Unit, University of Bari “Aldo Moro”, 70124 Bari, Italy; crescenziofrancesco.minervini@uniba.it

**Keywords:** ribosomal protein, Rpl22, *Drosophila*, DNA–protein interaction, transposable elements, heterochromatin, *Doc5/Porto1*

## Abstract

Chromatin is a highly dynamic biological entity that allows for both the control of gene expression and the stabilization of chromosomal domains. Given the high degree of plasticity observed in model and non-model organisms, it is not surprising that new chromatin components are frequently described. In this work, we tested the hypothesis that the remnants of the *Doc5* transposable element, which retains a heterochromatin insertion pattern in the melanogaster species complex, can be bound by chromatin proteins, and thus be involved in the organization of heterochromatic domains. Using the Yeast One Hybrid approach, we found Rpl22 as a potential interacting protein of *Doc5*. We further tested in vitro the observed interaction through Electrophoretic Mobility Shift Assay, uncovering that the *N-*terminal portion of the protein is sufficient to interact with *Doc5*. However, in situ localization of the native protein failed to detect Rpl22 association with chromatin. The results obtained are discussed in the light of the current knowledge on the extra-ribosomal role of ribosomal protein in eukaryotes, which suggests a possible role of Rpl22 in the determination of the heterochromatin in *Drosophila*.

## 1. Introduction

Chromatin [1] is a nucleoprotein complex that plays a key role in controlling cell behavior and chromosomal structure [2,3]. Its regulation is important in the control of cellular events, including genome packaging, replication, recombination, DNA repair, and transcription. The nucleosome, which comprises the four core histones (H2A, H2B, H3, H4), wrapped around with 168bp of DNA, and the linker histones H1 or H5 form the chromatosome, the structural unit of the chromatin [4].

Chromatin is found in two fundamental states during the cell cycle, the loosely condensed euchromatin and the highly compacted heterochromatin. A huge number of DNA–protein and protein–protein interactions contribute to the maintenance of these two structures, the plasticity of which is tightly regulated at the epigenetic level.

Many proteins act as structural components or regulators of the chromatin state, and post-translational modifications of many chromatin components play a fundamental role in maintaining the dynamic state of different chromatin domains. The ongoing ENCODE projects [5,6] aim to determine the nature of the epigenetic code and to what extent chromatin remodeling could influence the phenotypes.

Several pieces of observation suggest that ribosomal proteins (RPs) could have an active role in chromatin dynamics. First, RNA-mediated processes have a functional role in regulating chromatin structure and gene expression through the action of non-coding RNA molecules [7,8,9]. Second, a large fraction of the expressed lncRNA interacts with ribosomes in humans and mice (roughly 39% and 48%, respectively) [10].

Third, the presence of RPs in the nucleus is well-recognized since RPs are imported into the nucleus and assembled into pre-ribosomes in the nucleolus [11].

Therefore, a subset of RPs could be co-opted as chromatin components to perform additional functions under either physiological or exceptional conditions.

Heterochromatin is a partition of the eukaryotic genome, often regarded as useless and functionless. This concept is due to its low gene density and the consequent low impact of mutational load in this compartment on viability and fertility. The massive presence of satellite DNA and transposons in the constitutive heterochromatin has further reinforced this idea. However, since heterochromatin is associated with important functions and structures of the eukaryotic chromosomes, its role has been recently re-evaluated, both in model and non-model organisms. In *D. melanogaster*, several hundreds of genes have been mapped in the constitutive heterochromatin, thus demonstrating its importance in the physiology of cells, tissues, and organs in the fly [12], an observation largely supported by classic and modern genetics studies.

Several additional features make heterochromatin a fascinating genomic compartment. These include the massive presence of repeats and transposable elements, whose structural and functional roles remain elusive, despite decades of studies. In this respect, noteworthy examples still come from *D. melanogaster*. Several repeated loci have been characterized so far in the heterochromatin of *D. melanogaster*, and some of them play an extremely important role in determining critical phenotypes [13,14,15]. One of these relevant loci lies in the h39 region, a Hoechst-bright chromosomal band adjacent to the centromere of the second chromosome. Two well-studied satellite DNA sequences are clustered in the h39 region, the Responder locus (*Rsp*), and the *Bari1* repeat. The *Rsp* locus, in combination with the *Sd* euchromatic locus, constitutes the key components of one of the best-known segregation distortion systems [13]. The *Bari1* cluster is an array of roughly 80 copies of the *Bari1* transposon [16,17,18], depending on the fly strain [18,19] of the *Bari1* transposon. Elements of the *Bari1* family are *Tc1*-like transposons that have colonized the genome of several *Drosophila* species [17] and are active in the respective genomes [20,21].

The characterization of the *Bari1* copy number variation in several *D. melanogaster* populations [16,18,19] revealed that this is an extremely static array if compared to the closely linked *Rsp* locus [22,23]. Considering that the *Bari1* cluster origin probably dates back to the split of the melanogaster and simulans species, approximately 5 Mya, and that it is only present in *D. melanogaster* [16,17,24], it has been speculated that either it could be functionally connected to the *Rsp* locus or to other structural features of the h39 region. However, the presence of a small *Bari1* cluster on the X chromosome [17,25] and an additional small *Rsp* repeat on the third chromosome [26] and the highly repetitive nature of the h39 region complicate the molecular and genetic investigations of this chromosomal region.

Many transposon relics map at both sides of the *Bari1* cluster in the h39 region of the mitotic chromosomes of *D. melanogaster* [27]. A direct duplication of a 596 bp sequence identified upstream and downstream of the *Bari1* cluster is of particular interest. We hypothesized that this short duplication could be the signature of the transposition-mediated origin of the *Bari1* cluster (as also hypothesized for the minor X-linked *Bari1* cluster [25]). Alternatively, it could have a functional role in the h39 locus or in the heterochromatin [27]. Since the first hypothesis seems unreliable (due to the size incompatibility and outcome of the transposition event), in this work we tested the hypothesis that the above-described 596 bp sequence could have acquired a new function in the heterochromatin through the binding of a chromatin protein. Here, we present evidence that the ribosomal protein Rpl22 binds DNA in vitro, which suggests the possibility that it could be recruited as chromatin protein. The CG7434 gene, which encodes RpL22 protein, maps on the X chromosome. Three additional genes are present within the coding region of RpL22, two encoding snoRNAs (CR34590 and CR33918) and one encoding a ncoRNA (CR42491). This structure complicates the genetic analysis of the locus, and, in fact, no genetic studies have been performed focusing on this gene. At least two post-translational modification events have been characterized, involving phosphorylation of the Ser 289 and Ser290 residues of the RpL22 in *Drosophila* [28]. Among RPs, some members of the RpL22e family have unique structural features and several, apparently unrelated, possible functions. The *Drosophila* Rpl22 has additional Ala-, Lys- and Pro-rich sequences at the amino terminus, which resembles the carboxyl-terminal portion of histone H1 and histone H5 that have been demonstrated to be important in genome stability [29]. For this reason, it has been already hypothesized that Drosophila L22 might have two functions, namely, the role of DNA-binding similar to histone H1 and the role of organizing the ribosome [30]. Moreover, as hypothesized in previous works, any potential biological difference between Rpl22 and Rpl22-like proteins should be ascribed to the presence of the extra N-terminal domain of Rpl22, which can be the target of post-translational modifications [31].

We also have evidence that Rpl22 enters into the nucleus of different cell types, in addition to what was demonstrated previously in the male germline cells [32]. The possible implications in the stability of a specific heterochromatin region are discussed.

## 2. Materials and Methods

### 2.1. Plasmids Construction

The *Doc5* fragment flanking the *Bari1* cluster was PCR-amplified from the purified DNA of the BACR16M08 clone (described in [25]) using specific primers containing EcoRI adapters at the 5′ end. The PCR fragment was cloned into the EcoRI site of the pGEM-T vector (Promega) and verified by Sanger sequencing.

### 2.2. PCR Amplification

Primers used for PCR amplification are reported in Table 1.

### 2.3. One Hybrid Screening

The one hybrid screening was performed using the Matchmaker One-Hybrid System (Clontech, Kyoto, Japan) following the manufacturer recommendations.

A *Drosophila* embryonic cDNA library (cDNA pool from 0–21 h embryos of the Canton-s strain) in the pACT2 vector (Clontech) was used for the yeast one-hybrid screens.

The *Doc5* sequence was subcloned into the pHISi-1 vector at the EcoRI site and into the pLacZi vector. Both plasmids were linearized using either BamHI (pHISi-1) or NcoI (pLacZi) and transformed in the YM4271 *S. cerevisiae* strain using the TRAFO system [33]. Recombinant colonies, carrying the integrated constructs, were selected onto selective SD medium lacking either histidine (pHISi-1 vector) or uracil (pLacZi vector).

The background expression of the LacZ reporter was determined by a standard β-galactosidase assay. Colonies were transferred to Whatman filter paper discs and lysed with liquid nitrogen. Filters were then exposed to Z-buffer (Na_2_HPO_4_·7H_2_O 60 mM, NaH_2_PO_4_·H_2_O 40 mM, KCl 10 mM, MgSO4 1 mM, β–mercaptoethanol 50 mM, pH 7) containing X-gal (5-bromo-4-chloro-indolyl-b-D-galactopyranoside 0.33 mg/mL). Only clones without LacZ basal expression in 8 h were selected for further analyses. These clones were further transformed to integrate the linearized pHisi-1 vector. Background expression of the His cassette was found to be inhibited by 15 mM 3-AT.

### 2.4. Protein Expression and Purification

The plasmid sets used to express proteins in *E. coli* (pET/RpL22 for the full-length protein expression; pET/H5 for the H1-H5 domain expression; pET/L22 for the ribosomal domain expression) were constructed by PCR amplification of either the full-length, the 5′-terminal or the 3′-terminal part of the cDNA and subsequent cloning into the pET-200 vector.

Plasmids were transformed in chemically competent *E. coli* (BL21-DE3), and the cultures were induced with 1 mM IPTG at a cell density equivalent to 0.5 OD_600_ and maintained for 2.5 h at 37 °C. Cells were sonicated in 25 mM HEPES (pH 7.5), 1 M NaCl, 15% glycerol, 0.25% Tween 20, 2 mM β-mercaptoethanol, and 1 mM PMSF. A total of 10 mM imidazole (pH 8.0) was added to the soluble fraction before it was mixed with Ni-NTA resin (Qiagen, Hilden, Germany) according to the manufacturer’s recommendations. The resin was washed with sonication buffer containing 30% glycerol and 50 mM imidazole. Bounded proteins were eluted with sonication buffer containing 300 mM imidazole and dialyzed overnight against sonication buffer without imidazole. Purified proteins were analyzed on 12% SDS-polyacrylamide gel. Protein concentration was determined using the Protein Assay ESL Kit (Roche Basel, Switzerland).

### 2.5. Electrophoretic Mobility Shift Assay (EMSA)

In total, 5 μg of the pT/Doc5 plasmid was EcoRI-digested and the released fragment was gel-purified using the QIAquick Gel Extraction Kit (Qiagen). A filling-in reaction was performed to end-label the target DNA. A total of 50 ng of the eluted fragment was incubated with [α^32^P]ATP (Perkin Elmer, Waltham, MA, USA), 1X Klenow reaction buffer and 2U of Klenow fragment (Roche, Basel, Switzerland).

Labeled fragments were purified using Sephadex G50 exclusion chromatography columns.

A total of 2 ng of the labeled fragment was incubated with the appropriate protein (either the full-length Rpl22, the H1-H5 domain or the ribosomal domain) in binding buffer as described in [34] (25 mM HEPES, pH 7.6, 50 mM NaCl, 1 mM EDTA, 1 mM DTT, 0.1 mg/mL BSA, 2.5 mM spermidine, 10% glycerol, and 0.1 mg/mL poly (dI-dC). Competition experiments were performed using either linear pUC19 (SmaI linearized) or sonicated λ phage DNA (200–1000 bp size range enrichment). The binding reaction was started by adding the protein extract and incubated for 20 min at 25 °C, then loaded directly onto 5% polyacrylamide (75:1 acrylamide:bisacrylamide) pre-run gel in 40 mM Tris–acetate, 2.5 mM EDTA (pH 7.8). Gels were run for 4.5 h at 4 °C at 10 V/cm and dehydrated using a gel-dryer. DNA–protein complexes were visualized by autoradiography using a STORM phosphorimager (Molecular Dynamics).

### 2.6. Fluorescence In Situ Hybridization and Immunofluorescence on Polytene Chromosomes

Fluorescence in situ hybridization experiments on polytene chromosomes were performed as described in [35]. Polytene chromosomes were prepared from third instar larvae of *D. simulans* and *D. sechellia*, reared on standard cornmeal medium at 18 °C. Salivary glands were dissected in PBS using a pair of dissection needles, fixed in 40% acetic acid, and squashed onto microscopy slides. Probes were labeled using the nick translation method with Cy3-dUTP, hybridized overnight at 37 °C.

Digital images were obtained using an Olympus epifluorescence microscope equipped with a cooled CCD camera. Gray scale images, recording Cy3 and DAPI fluorescence, were obtained separately using specific filters and were pseudo colored and merged to obtain the final image using the Adobe Photoshop software.

Immunodetection experiments of Rpl22 and fibrillarin on polytene chromosomes of the Oregon-R (wild type) were performed according to James et al. [36] using the polyclonal primary anti-Rpl22 antibody (diluted 1:50) raised in rabbit (Invitrogen Carlsbad, CA, USA, Minervini et al. submitted) and the monoclonal (G-8sc-374022 Santa Cruz Biotechnology Inc., Dallas, TX, USA) anti-fibrillarin antibody raised in mouse. An FITC (fluorescein isothiocyanate)-conjugated anti-rabbit Ig (whole antibody) raised in sheep (diluted 1:20) and the Alexa Fluor 488 goat anti-mouse antibody (Life Technologies, Carlsbad, CA, USA, 1:200 dilution) were used as secondary antibodies. Following incubation, the slides were washed three times in PBS, stained with DAPI (4,6-diamidino-2-phenilindole) at 0.01μg/mL and mounted in anti-fading medium. Immunodetection on S2R+ cells were performed as previously described in [20,21] using the above-described antibodies.

### 2.7. Other Methods

Sequencing of the cloned fragments was performed at the BMR Genomics sequencing facility (Padova, Italy).

Global alignments were performed using DNA Strider [37]. Local alignments were performed using BLAST at the NCBI website.

NLS signals were searched with cNLS Mapper (http://nls-mapper.iab.keio.ac.jp/cgi-bin/NLS_Mapper_form.cgi (accessed on 1 March 2021)) [38] using a cutoff score = 7 in the entire protein sequence, and with Nucpred (https://nucpred.bioinfo.se/cgi-bin/single.cgi (accessed on 2 March 2021)) [39].

## 3. Results

**Figure 1:**
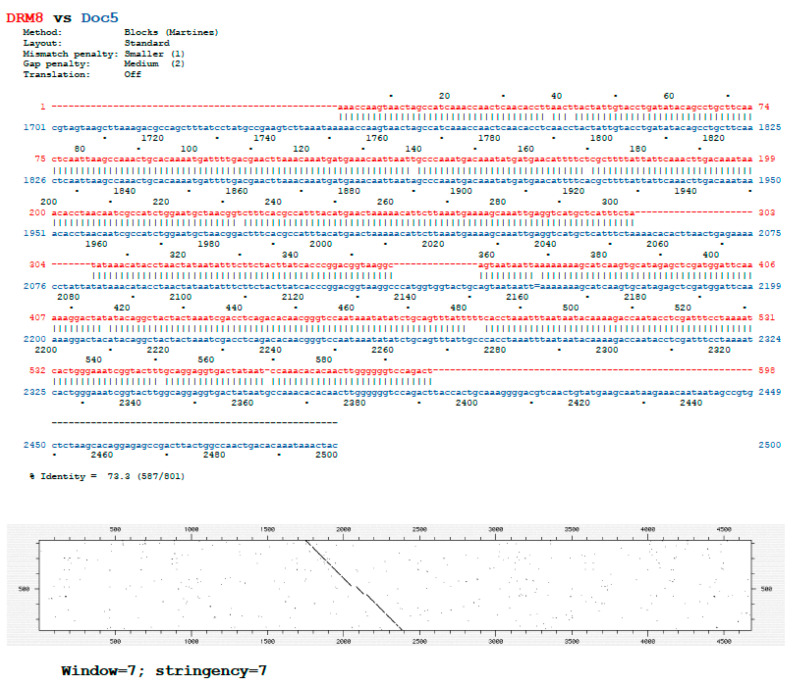
Comparison of the *Doc5* reference sequence and the 596 bp sequence identified at both sides of the *Bari1* cluster in the h39 region of the chromosome 2 of *D. melanogaster*. The global sequence alignment and the dot-plot comparison are shown.

We have previously identified a 596 bp DNA sequence duplication (formerly named DRM8) at both sides of the *Bari1* cluster in the heterochromatin of 2R chromosome of *D. melanogaster* [27]. Specifically, this repetitive sequence maps in the h39 region, and it has been proven lately to be a remnant of the *Doc5*/*Porto1* element, a highly repeated non-LTR retrotransposon in the heterochromatin of *D. melanogaster* [40]. The similarity between the DRM8 sequence and the reference *Doc5*/*Porto1* element is shown in Figure 1. Hereafter, we will refer to this sequence as *Doc5*.

Several copies of the *Doc5* can be found in the reference genome of *D. melanogaster* (see Table 2). In silico analyses reveal that *Doc5* maps exclusively in the constitutive heterochromatin of the two major autosomes of *D. melanogaster*, including the centromere, as well as at the eu-heterochromatin transition.

**Figure 2:**
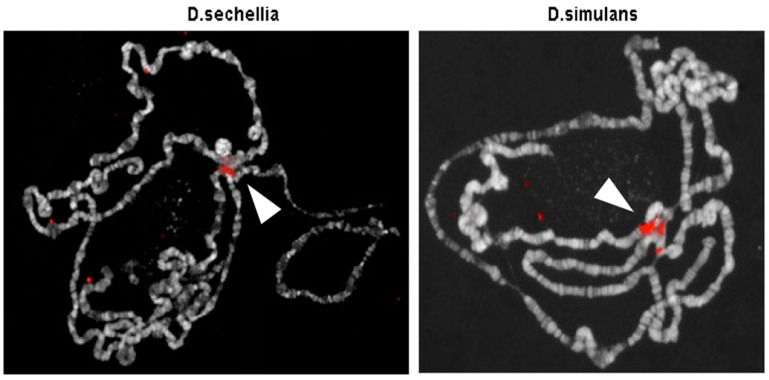
The distribution of the *Doc5* transposon was analyzed by FISH in the genome of *D. sechellia* (**left panel**) and *D. simulans* (**right panel**), two species closely related to *D. melanogaster*. The *Doc5* fragment cloned from the h39 region (596bp sequence) was used as probe. Arrowheads point to the chromocenter.

The heterochromatic localization of the *Doc5* element is also a conserved feature in closely related species of the melanogaster complex, such as *D. simulans* and *D. sechellia*, as demonstrated by the results of FISH experiments on polytene chromosomes (Figure 2).

The hybridization signals in the chromocenter and at the eu-heterochromatin transition on the chromosome arms (Figure 2) clearly highlight a heterochromatin-specific pattern of *Doc5,* which is conserved in *D. simulans* and *D. sechellia*. The positional conservation of a transposon relic might indicate its possible functional or structural role, such as the determination of the chromatin identity domains or the implication in transcriptional processes.

The evolutionary conservation of the heterochromatic pattern and the high degree of sequence identity of the *Doc5* fragment duplicated at both sides of the *Bari1* cluster prompted us to hypothesize a possible structural role of the *Doc5* sequence both in the heterochromatin of *D. melanogaster* and in the identity of the h39. It was previously suggested that the preservation of a repetitive non-coding DNA sequence, especially in the heterochromatin, could be promoted with the aid of stabilizing binding proteins [41], such as chromatin proteins. To test this hypothesis, we performed a One-Hybrid System assay aimed at the identification of proteins that potentially interact with the *Doc5* fragment.

The double selection method (i.e., His prototrophy and positivity to the β-galactosidase test) applied to identify positive clones ensures that the false positive rate is minimized.

Twenty-four positive clones, selected on selective media lacking histidine, were further tested with the β-galactosidase activity (Table S1). Many of these clones turned rapidly blue upon β-galactosidase testing (3–5 h). However, a large fraction (46%) of such clones matched to Rpl22 transcripts after Sanger sequencing and BLASTN analysis. Based on the fastness of color turning (the smallest the better) and the relative abundance of positive clones, we chose Rpl22 as the candidate for further investigations.

The positive clones obtained from the first round of screening were further validated by independent transformation of the isolated plasmids into the bait-containing yeast strains (i.e., yeast strains containing the *Doc5*-His and *Doc5*-lacZ cassette, data not shown) to confirm the bait–pray interaction.

To further solidify this result, we assayed the Rpl22–*Doc5* interaction in vitro. The Rpl22 protein was expressed and purified in *E. coli* and used for in vitro binding assays in order to test its ability to bind an end-labeled *Doc5* fragment (see Materials and Methods).

**Figure 3:**
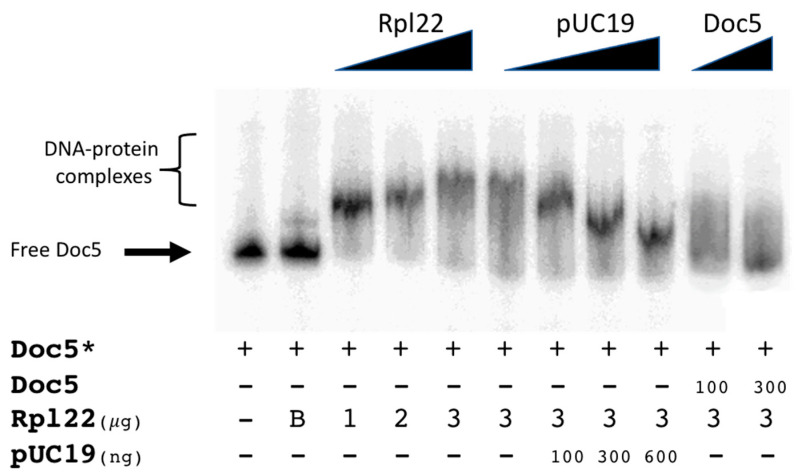
The binding of Rpl22 to *Doc5*. The amount of labeled fragment (Doc5 *, Figure 3) in each lane is 3 ng. The amounts of unlabeled specific competitor (ng of Doc5), Rpl22 (μg), and unlabeled non-specific competitor (ng of linearized pUC19) are indicated in the figure legend under the respective lanes. Increasing amounts of purified Rpl22 protein (lanes 3–5) and non-specific (lanes 6–9) and specific (lanes 10–11) competitors are indicated on the top by triangles. A negative control (lane 2) was performed following the incubation of the Doc5-labeled probe with 3 µg of non-induced *E. coli* (BL21 strain) lysate (indicated with B). The labeled fragments are indicated with an asterisk (*).

As can be observed in Figure 3 (lanes 3–5), increasing amounts of purified protein led to a slower migration in a polyacrylamide gel, suggesting the formation of progressively slower DNA–protein complexes. In our hands, 3 µg of the protein extract led to the formation of the slowest DNA–protein complex. This pattern could be explained by either the presence of multiple binding sites in the target or by the possible formation of multimeric protein complexes that bind the target fragment.

The observed protein binding is specific and reversible, as demonstrated by the competition assays in Figure 3. While a 200-fold amount of unspecific competitor is not sufficient to disrupt the Rpl22–*Doc5* interaction (Figure 3, lanes 6–9), a 30-fold amount of target fragment completely disrupts the observed DNA–protein binding (Figure 3, lanes 10–11). Additional controls to assess the specificity of the binding were performed using either an unrelated DNA fragment, or using a different non-specific competitor DNA (Figure S1).

**Figure 4:**
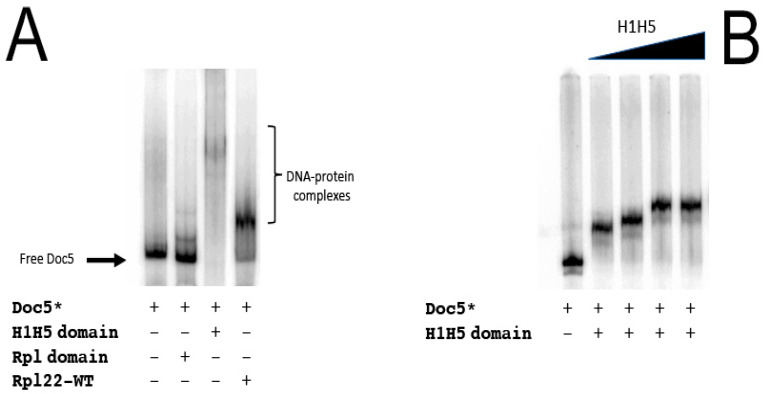
Dissection of the DNA-binding domain of Rpl22 in vitro. Labeled fragments are indicated with an asterisk (*). (**A**) EMSA analysis of the ribosomal and the histone-like domains of Rpl22. (**B**) EMSA analysis of the histone-like domain. A total of 3 µg of the Rpl22 (WT) and 1.5 µg of the H1-H5 and ribosomal domains were used to maintain the unaltered DNA:protein molar ratio. A schematic representation of the two main domains of Rpl22 protein is depicted at the top of the figure. Asterisk indicates that the fragment is labelled.

We next investigated whether the two domains of Rpl22 could differentially contribute to the observed DNA–protein interaction. The H1-H5 domain and the ribosomal domain were independently tested in EMSA assays for their ability to interact with *Doc5*. As can be observed in Figure 4, only the H1-H5 domain retains the ability to bind the *Doc5* fragment tested (Figure 4, lane 3), whereas the ribosomal domain does not (Figure 4, lane 2) if compared to the binding observed for the wild-type Rpl22 protein (Figure 4, lane 4). Similar to what observed for the wild-type protein (Figure 3, lanes 3–5), the H1–H5 domain interacts with the *Doc5* sequence in a dose-dependent manner (Figure 4B).

To further investigate the possible role of Rpl22 in the chromatin dynamics, we tested the Rpl22 protein localization in both *D. melanogaster* cultured cells, in order to check whether the protein co-localizes with chromosomes. We performed immunofluorescence localization of the native Rpl22 protein on polytene chromosomes of the Oregon-R wild-type strain and inn cultured S2R+ cells, using a polyclonal antibody raised against the Rpl22 protein.

**Figure 5:**
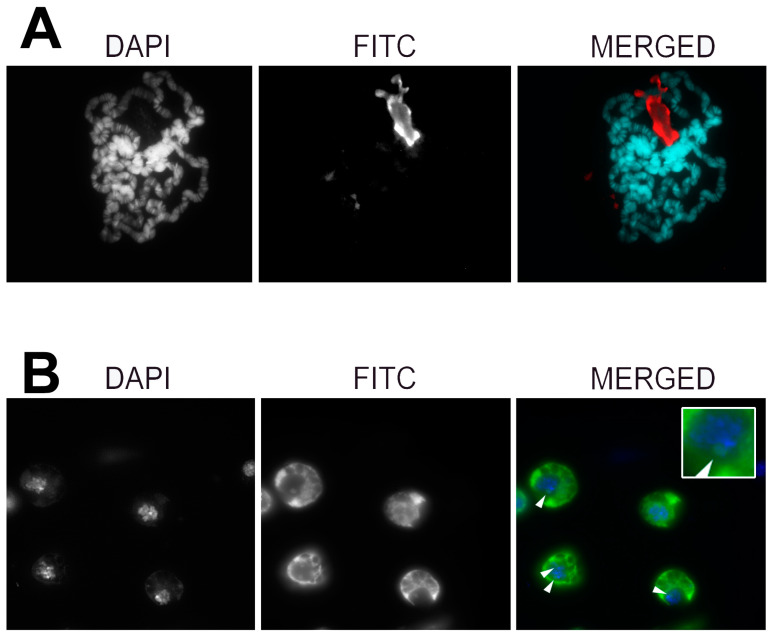
Pattern of subcellular immunolocalization of Rpl22 in *D. melanogaster* salivary gland nuclei (**A**) and in cultured S2R+ cells (**B**). White arrowheads point to nucleoli. A magnified detail of the nucleolar co-localization is reported in the inset. Additional details on the localization of Rpl22 to nucleoli are given in Figure S2.

The results obtained (Figure 5) clearly show that Rpl22 localizes to the nuclei, with a marked nucleolar localization that has been further confirmed by co-localization with the nucleolar marker fibrillarin, (Figure S2) both in salivary gland cells (Figure 5A) and in cultured cells (Figure 5B), without any additional evidence of localization to chromatin.

In silico prediction of the nuclear localization of Rpl22 using cNLS Mapper [38] suggests its nuclear localization, with the best scoring NLS signal (score 7/7) mapped at position 234. A similar search, using NucPred [39] as an alternative algorithm, returned the sequence GKGQKKKK (position 181, score 0.28; a 0.30 threshold corresponds to 77% sensitivity and 55% specificity).

In the absence of additional experimental evidence, the possible role of Rpl22 in the heterochromatin can be inferred from interactomic data obtained in previously published works. Out of the ninety-one RpL22-interacting proteins that are annotated in FlyBase, 13 are non-RPs. Notably, 12 out of the 13 interacting proteins are not directly linked with the translational machinery.

Rpl22 interacts with protein involved in heterochromatin organization (vig and vig2 [42,43]), piRNA biogenesis (Fmr1 and its associated miRNA, bantam [44]), and transcriptional repression (Ago1 and Ago2 [42]) (reported in Table 3). Such interactions further suggest the involvement of Rpl22 in chromatin determination and transcriptional pathways, supporting our hypothesis.

## 4. Discussion

The stabilization of large chromosomal domains containing extended repeat blocks essentially depends on the chromatin architecture that wraps these loci. Both the Encode [5] and modEncode [45] projects have had a leading role in the determination of the genome-wide chromatin status in *H. sapiens* and model organisms, respectively. The outcome of these huge projects led to the birth of epigenomics that aims to link cell-type-specific gene expression to chromatin structure. The specific features of chromatin domains are also of critical importance for genome evolution since the propensity of certain loci to be converted and relocated from the euchromatin to the heterochromatin is probably determined by the ancestral epigenetic marks [46]. For this reason, profound knowledge of chromatin dynamics is fundamental in the determination of the evolutionary trajectories that chromosomes follow.

However, chromatin is a highly dynamic biological entity, and for this reason, it is difficult to provide a definitive and exhaustive description. Unbiased approaches, i.e., not focused on a particular developmental stage or specific tissue, allow for a near-to-complete characterization of chromatin-associated proteins. It follows that the elucidation of the changing state of chromatin in the most diverse cellular types is of particular importance toward the complete understanding of physiological and pathological conditions [47].

Here, we report that a ribosomal protein binds the *Doc5* transposon, a non-autonomous TE family enriched in the heterochromatin of *D. melanogaster* and closely related species [48], providing in vitro experimental evidence for a functional interaction of Rpl22 with DNA, and possibly to chromosome and chromatin. In *Drosophila*, the direct binding of protein to TEs, especially involving retrotransposons, has been previously reported [49,50,51]. In a yeast one-hybrid assay, we probed a *D. melanogaster* expression library with *Doc5* as bait and found Rpl22 as the best candidate interacting protein. We have further validated the DNA–protein interaction with a series of EMSA experiments that confirmed the results of the experiments in yeast. We further demonstrated that the NH-terminal domain (H1–H5 domain) of the protein is both necessary and sufficient to bind DNA. Furthermore, the assays performed in vitro show that the *Doc5*–Rpl22 interaction depends on the amount of protein input. We cannot dismiss the hypothesis that this behavior could depend both on the presence of multiple binding sites on the target (which we have not investigated), and on the ability of Rpl22 to multimerize or to form homogeneous aggregates. In addition, the net charge density of the expressed H1-H5 domain is greater than that of the wild-type Rpl22 protein (27.14/15.8 KDa vs. 36.51/30.6 KDa, respectively, at pH = 7), which can account for the increased shift of the H1-H5/*Doc5* complex if compared to the wild-type Rpl22/*Doc5* complex (Figure 4).

What is the relevance of our findings? Our results let us hypothesize that Rpl22 could have a potential role in the organization of chromatin, possibly in heterochromatin, and this hypothesis is supported by several studies reporting that RPs are linked to biological processes occurring in the nucleus [52]. RPs have been found associated at transcription sites in *Drosophila* polytene chromosomes. This unexpected finding suggested that ribosomal subunits could be associated with nascent mRNAs [53]. An additional study in *Saccharomyces cerevisiae* showed that RPs bind to noncoding RNA genes, suggesting that the RPs–RNA association might be independent of the translatability of the transcript and might involve free RPs that are not assembled into ribosomes [54]. Several other examples of RPs with extra ribosomal functions at transcription sites have been reported to date. Some RPs auto-regulate their expression by affecting translation, splicing, or transcription by interacting with their mRNA, or promoter [55,56,57]. RPs are also able to interact with transcription factors at the promoters of genes. RpL11 binds the oncoprotein c-MYC at the promoter of c-MYC target genes [58,59], RpS3 is a subunit of the NF-κB DNA-binding complex involved in chromatin binding and transcription regulation of specific genes [60]. RpS3 phosphorylation at serine 209 by IKKb is crucial for RPS3 nuclear localization in response to activating stimuli [61].

Rpl22 is a ribosomal protein with a prevailing cytoplasmic localization. Past-published reports claimed that Rpl22 also localizes to the nucleus of *Drosophila* cells. Ni and collaborators [62] demonstrated that Rpl22 expressed at endogenous levels localizes in the nucleus of *Drosophila* Kc (embryo-derived) and cl-8 (derived from imaginal discs) cell cultures, and it is associated with chromatin, resulting in gene suppression. Immunofluorescent staining and chromatin immunoprecipitation (ChIP) analyses demonstrated that RpL22 and H1 are both associated with condensed chromatin. In the same study, it was demonstrated that the overexpression of RpL22 caused the transcriptional repression of two-thirds of the genes suppressed by histone H1. By contrast, RpL22 depletion caused the up-regulation of the transcription of several tested genes, supporting a role for RpL22 as transcriptional repressor [62]. These observations imply the involvement of Rpl22 in global transcriptional processes.

However, Rpl22 has not been previously identified in surveys aimed at the identification of chromatin structure. This can be due to an experimental bias when searching histone modifications [63]. On the other hand, unbiased studies have been focused on euchromatic genomic regions only [64]. Conversely, our approach was based on the search of proteins that interact with a heterochromatic sequence, and our results support a role of Rpl22 in the chromatin. To what extent Rpl22 could participate in the determination of chromatin domains remains to be determined. Another potential implication of our findings concerns the possible role of the non-autonomous *Doc5* transposon in the *D. melanogaster* genome. Non-autonomous TEs often acquire new functions in complex genomes, over evolutionary time. Many examples of evolutionarily inactive TEs that have been co-opted, exapted, or domesticated are described in the scientific literature [65,66].

It has been demonstrated that *Doc5* is under the control of the piRNA pathway [67,68]. Since no potentially active *Doc5* copies are found, these findings suggest that the short RNAs generated from *Doc5* could have alternative roles in the regulation of gene expression, or alternatively in the regulation of the transposition of other, unrelated, TEs.

Alternatively, *Doc5* could mark a chromatin domain with a structural function that prevents the excessive expansion of the *Bari1* cluster. This hypothesis could be extended to other species-specific heterochromatic repeats, since the *Bari1* cluster is unique to the *D. melanogaster* species, while *Doc5* is present in the genome of sibling species [17].

In contrast with previously reported results, we were not able to demonstrate/reproduce a pan-nuclear localization of Rpl22 in S2R+ cells. Our experiments only revealed a nucleolar localization of the protein, without any detectable association to chromatin. This contrasting result could be explained considering the limitations of the immunolocalization technique, which would not allow for the detection of a small amount of chromatin proteins. Moreover, the differences between the cell lines used in our experimental setup (S2R+) and in previous studies (Kc) should be taken into account. Kc are male derived, while S2R+ derive from females. Kc have a plasmatocyte-like phenotype, while S2R+ combines properties of plasmatocytes and crystal cells [69]. Finally, Kc and S2R+ have different ploidy, since Kc are approximately 4n, while S2R+ are 2.5n [70]. We can, therefore, hypothesize that the nuclear localization and the association to chromatin is cell-type dependent. Consequently, we cannot dismiss the fact that, in S2R+ cells, Rpl22 can also enter the nucleus under particular experimental conditions. An additional limitation of our study is the lack of confocal microscopy analyses, which grants a powerful resolution at the subcellular level.

Similarly, we did not detect Rpl22 signals on the polytene chromosome arms, nor in the chromocenter. The limitation in terms of resolution using the polytene chromosome to assess the presence of DNA–protein interactions in heterochromatin is due to the under-replicated nature of the chromocenter [71,72]. Moreover, it has been demonstrated that Rpl22 is subjected to post-translational modifications in testis [31]. SUMOylation, phosphorylation, and possibly other unexplored post-translational modifications could also affect the Rpl22 localization and its ability to be engaged in additional functions, other than translation. Post-translationally modified Rpl22 could potentially exit from the nucleolus and associate to chromatin in particular, unexplored, physiological conditions or in response to environmental stresses. This change in localization could be elicited by protein post-translational modification, as demonstrated in previous studies involving Rpl22 [31].

Despite the lack of localization to chromosomes and chromatin, several additional observations support the hypothesis of an involvement of Rpl22 in chromatin dynamics. There are 12 out of 91 Rpl22-interacting proteins that suggest its involvement in chromatin-related processes. Furthermore, RpL22 has been identified as one of the two hundred genes required for mitotic spindle assembly in *Drosophila* S2 cells in an RNAi screen [73], and the down-regulation of the RpL22 gene also results in aberrantly short, monopolar spindles in S2 cells. These data together with the demonstration of the DNA binding ability of Rpl22 presented in this paper, offer a new perspective of how Rpl22 could participate in chromatin dynamics, at least under specific conditions that has yet to be determined. Additionally, previous genome-wide ChIP-on-chip analysis in the fission yeast *S. pombe* revealed the presence of ribosomal protein complexes at transcription sites with unexpected peaks at centromeres, raising the intriguing hypothesis that RP complexes are involved in tRNA biogenesis and possibly centromere functions [57].

## 5. Conclusions

We have presented in vitro evidence of the interaction between a typical heterochromatic sequence and a ribosomal protein in *D. melanogaster*. However, experiments in vivo do not confirm the results of experiments in vitro, suggesting that further investigation is needed to reveal the physiological role of Rpl22 in the context of the chromosome structure.

While further studies are needed to understand if the *Doc5* element has been co-opted to absolve further functions in the heterochromatin, several suggestive hypotheses could be proposed. *Doc5* could act as a bidirectional promoter that allows for the transcription of the *Bari1* cluster in order to activate the piRNA-mediated repression of the transposition. In this hypothesis, the ribosomal protein Rpl22 could help in the transcriptional activation from this promoter [74] or in the stabilization of non-coding RNAs [54]. Considering that the *Bari1* elements tested so far are transpositionally active [20,21,75] and are capable of autonomous transcription [76,77], this hypothesis could at least partially explain the low transposition activity observed in *D. melanogaster* laboratory strains [20]. In a companion paper, Minervini et al. (Minervini et al. submitted to *Genes*) also demonstrated that Rpl22 binds in vitro to a transposable element-derived consensus sequence. This observation leads to the hypothesis that ribosomal proteins could be also involved in controlling the activity of TEs in *Drosophila*, not only at the translation level [78] but also at the transcriptional level.

## Figures and Tables

**Table 1:** List of primers used in this study.

Primer	Sequence	Usage
ADread	5′-CTATTCGATGATGAAGAT-3′	sequencing
pACT2seq	5′-TACCACTACAATGGATG-3′	sequencing
pACT2 up	5′-CTATTCGATGATGAAGATACCCCACCAAACCC-3′	Amplification/cloning
pACT2 low	5′-GTGAACTTGCGGGGTTTTTCAGTATCTACGAT-3′	Amplification/cloning
His1_up	5′-GAGGCCCTTTCGTCTTCAA-3′	Amplification/cloning
His1_low	5′-CTAGGGCTTTCTGCTCTGTCATCT-3′	Amplification/cloning
Doc5_up	5′-ACGGCTATTATTGTTTCTTATTGCT-3′	Amplification/cloning
Doc5_low	5′-TTATCCTCATCCCTTATCCTATGT-3′	Amplification/cloning
pETup	5′-CACCATGGCTTACCCATA-3′	Amplification/cloning
pETlow	5′-ATAAAAGAAGGCAAAACGATG-3′	Amplification/cloning
H5low	5′-CTAACGCAGCACGTTCTTCTT-3′	Amplification/cloning
L22up	5′-CACCAAGGTGGTCAAGAAGAA-3′	Amplification/cloning

**Table 2:** The distribution of the *Doc5* transposon in the *D. melanogaster* genome.

Subject Accession	Start	End	Chromosome	% Identity	Alignment Length	Evalue	Bit Score	Chromosome Map Position
NT_033779.5	23,430,152	23,429,509	2L	85.891	645	0	643	h35–36
NT_033779.5	23,037,532	23,037,717	2L	91.237	194	2.03 × 10^−67^	257	h35–36
NW_001845128.1	3990	4435	2CEN	100	446	0	824	deep het
NW_001845128.1	3875	3990	2CEN	100	116	1.24 × 10^−54^	215	deep het
NW_001844967.1	11,017	10,572	2CEN	100	446	0	824	deep het
NW_001844967.1	11,132	11,017	2CEN	100	116	1.24 × 10^−54^	215	deep het
NW_007931075.1	7023	6491	2CEN	82.655	565	2.43 × 10^−121^	436	deep het
NW_007931075.1	9914	9382	2CEN	82.655	565	2.43 × 10^−121^	436	deep het
NT_033778.4	396,636	397,234	2R	99.332	599	0	1083	h41–h44
NT_033778.4	165,422	164,833	2R	95.326	599	0	942	h41–h44
NT_033778.4	74,075	74,637	2R	92.833	586	0	824	h41–h44
NT_033778.4	298,134	297,689	2R	100	446	0	824	h41–h44
NT_033778.4	872,342	871,810	2R	82.655	565	2.43 × 10^−121^	436	h41–h44
NT_033778.4	875,233	874,701	2R	82.655	565	2.43 × 10^−121^	436	h41–h44
NT_033778.4	1,413,093	1,413,384	2R	91.333	300	2.47 × 10^−111^	403	h45
NT_033778.4	3,518,328	3,518,018	2R	88.179	313	1.95 × 10^−97^	357	h41–h44
NT_033778.4	5,012,337	5,012,064	2R	82.818	291	4.43 × 10^−59^	230	h46
NT_033778.4	298,249	298,134	2R	100	116	1.24 × 10^−54^	215	h41-h44
NT_033778.4	5,012,596	5,012,448	2R	84.302	172	4.59 × 10^−34^	147	h46
NT_037436.4	24,877,579	24,878,162	3L	87.081	596	0.0	656	h49
NT_037436.4	24,914,416	24,913,834	3L	86.745	596	0.0	645	h49
NT_037436.4	24,944,076	24,944,602	3L	87.199	539	2.93 × 10^−170^	599	h49
NT_037436.4	24,461,859	24,462,331	3L	85.443	474	6.71 × 10^−127^	455	h47
NT_037436.4	23,664,082	23,663,846	3L	94.583	240	9.00 × 10^−101^	368	80F9
NT_037436.4	23,663,844	23,663,639	3L	92.754	207	1.20 × 10^−79^	298	80F9
NT_037436.4	25,490,094	25,490,271	3L	94.382	178	2.02 × 10^−72^	274	h49–h50
NT_037436.4	27,913,043	27,913,159	3L	93.277	119	7.57 × 10^−42^	172	h51
NT_037436.4	24,502,780	24,502,891	3L	92.035	113	2.12 × 10^−37^	158	h48
NT_037436.4	27,912,886	27,913,038	3L	81.609	174	2.15 × 10^−27^	124	h51
NT_033777.3	646,928	646,337	3R	86.612	605	0.0	649	h54–h56
NT_033777.3	4,042,323	4,042,066	3R	94.961	258	6.86 × 10^−112^	405	81F
NT_033777.3	1,401,453	1,401,023	3R	83.991	431	1.50 × 10^−103^	377	h54–h56
NT_033777.3	4,050,664	4,050,455	3R	95.238	210	3.28 × 10^−90^	333	81F
NT_033777.3	3,992,575	3,992,365	3R	94.787	211	1.53 × 10^−88^	327	81F
NT_033777.3	4,039,954	4,039,769	3R	91.710	193	1.57 × 10^−68^	261	81F
NT_033777.3	2,453,123	2,453,400	3R	80.357	280	4.53 × 10^−44^	180	h56
NT_033777.3	2,453,399	2,453,508	3R	92.793	111	5.90 × 10^−38^	159	h56
NW_001845051.1	2554	2831	UNK	80.357	280	4.53 × 10^−44^	180	deep het
NW_001845051.1	2830	2934	UNK	89.189	111	1.29 × 10^−29^	132	deep het

The *Doc5* sequence (596 bp) was used as a query in BlastN analyses against the *D. melanogaster* reference genome (Release 6). The approximate map positions in the rightmost column were inferred by comparison with the data in [12]. Only alignments longer than 100 bases are shown. Deep het: deep heterochromatin. UNK: unknown map position.

**Table 3:** Rpl22 interacting proteins involved in heterochromatin functions. Information retrieved from Flybase (last accessed August 2021).

Gene Name	FlyBase ID	Function	Inferred by	Reference
vig	FBgn0024183	Heterochromatin organization	Co-IP	[42]
AGO1	FBgn0262739	transcriptional repression	Co-IP	[42]
AGO2	FBgn0087035	transcriptional repression	Co-IP	[42]
vig2	FBgn0046214	Heterochromatin organization	Mass-spec	[43]
Fmr1	FBgn0028734	piRNA biogenesis	Co-IP	[42]
ban	FBgn0262451	piRNA biogenesis	Co-IP	[42]
esi2	FBgn0285992	Unknown	Co-IP	[42]
smt3	FBgn0264922	mitosis	Co-IP	[31]

## Data Availability

Not applicable.

## Supplementary Materials

The following are available online at https://www.mdpi.com/article/10.3390/genes12121997/s1, Table S1: List of the yeast clones tested in the β-Galactosidase assay. Supplementary Figure S1: In vitro assays (EMSA) suggesting the specificity of the Doc5/Rpl22 binding. Supplementary Figure S2: (**A**). Rpl22 co-localizes with fibrillarin in S2R+ cells. From the left to the right: DAPI, anti-fibrillarin, anti-Rpl22, merged signals. Signal pseudo-coloring in the merged image is as follows. DAPI: blue; Fibrillarin: red; Rpl22: green. The arrowhead in the merged image point to the nucleolus. A magnified detail of the nucleolar co-localization is reported in the inset. (**B**). Rpl22 co-localizes with fibrillarin in polytene nuclei. From the left to the right: DAPI, anti-fibrillarin, anti-Rpl22, merged signals. Signal pseudo-coloring in the merged image is as follows. DAPI: blue; Fibrillarin: green; Rpl22: red. Arrowheads in the merged image point to nucleoli. Table S1. List of the yeast clones tested in the Galactosidase assay. Clones carrying Rpl22 sequences are reported in bold.
