# Supplementary material for: Evidence of the Physical Interaction between Rpl22 and the Transposable Element Doc5, a Heterochromatic Transposon of Drosophila melanogaster
Source: Genes (Basel). 2021 Dec 16;12(12):1997. doi: 10.3390/genes12121997 (PMC8701128; doi:10.3390/genes12121997)
Supplement: Supplementary file 1 [file genes-12-01997-s001.zip › genes-1474839 - Supplementary.pdf]

Supplementary Figure S1

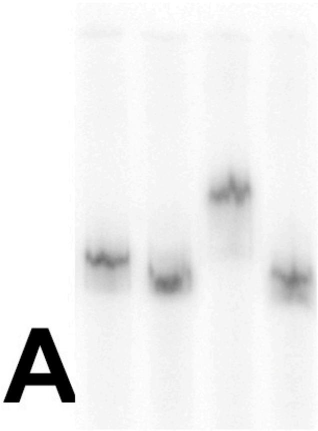

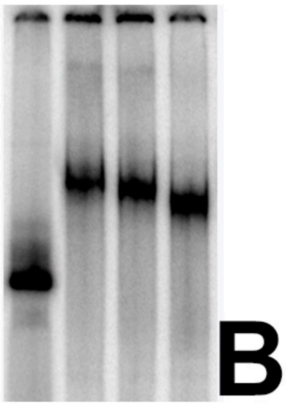

Figure S1. In vitro assays (EMSA) suggesting the specificity of the Doc5/Rpl22 binding.

Panel A. Lane 1: free end-labelled Doc5; lane 2: free end-labelled 550 bp fragment amplified from the yellow gene; lane 3: Doc5 incubated with 3ug of Rpl22; lane 4: 550 bp fragment amplified from the yellow gene incubated with 3 ug of Rpl22. Panel B. Increasing amount of sonicated lambda DNA (lanes 3 and 4, 300 ng and 1,5 ug respectively) was used as cold competitor DNA to disrupt the Doc5/Rpl22 binding. Lane 1: free end-labelled Doc5: lane 2: Doc5 incubated with 3ug of Rpl22 (no competitor).

Supplementary Figure S2

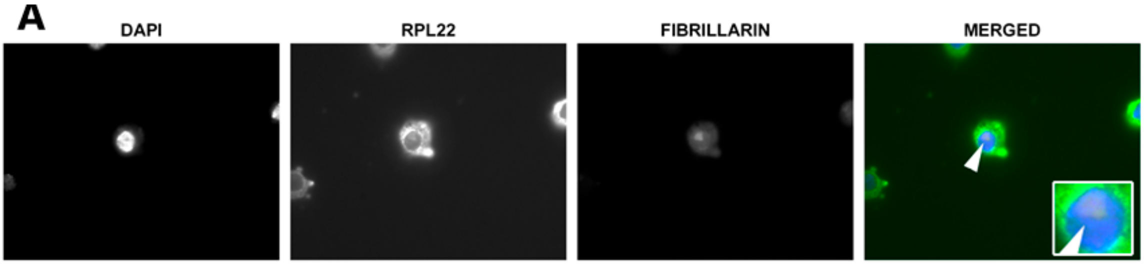

Figure S2.A. Rpl22 co-localizes with fibrillarin in S2R+ cells. From the left to the right: DAPI, anti-fibrillarin, anti-Rpl22, merged signals. Signal pseudo-coloring in the merged image is as follows. DAPI: blue; Fibrillarin: red; Rpl22: green. The arrowhead in the merged image point to the nucleolus. A magnified detail of the nucleolar co-localization is reported in the inset.

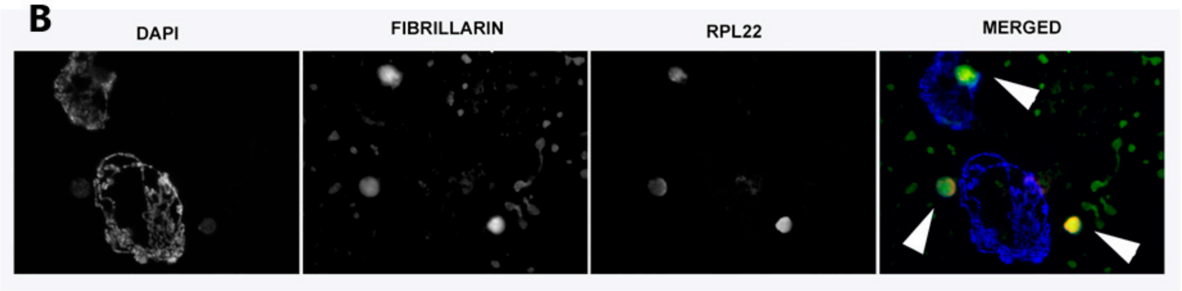

Figure S2. B. Rpl22 co-localizes with fibrillarin in polytene nuclei. From the left to the right: DAPI,

anti-fibrillarin, anti-Rpl22, merged signals. Signal pseudo-coloring in the merged image is as follows. DAPI: blue; Fibrillarin: green; Rpl22: red. Arrowheads in the merged image point to nucleoli.

Supplementary Table S1

Table S1. List of the yeast clones tested in the β–Galactosidase assay. Clones carrying Rpl22 sequences are reported in bold.

clone	Test βGal (h)	BLAST HIT(s)
1.1	5	**RpL22**
2.1	ND	No insert
2.2	5	RpL13a*
2.4	*ND*	CG16971
2.5	7	RpS7
2.6	3	CG7172-RA
4.1	3	**RpL22**
4.2	4	**RpL22**
5.1	3	**RpL22**
5.4bis	3	RpL10e
7.1	5	**RpL22**
8.1	O/N	CG14112 (SNCF)
8.2	5	CG18269
8.3bis	3	**RpL22**
8.4	3	Failed
8.5	3	Failed
8.6	4	**RpL22**
10.1	3	**RpL22**
10.3	5	RpS14a*
10.4	5	RpL12
11.2	ND	Rpa1
12.1	3	**RpL22**
13.1	3	csw CG3954-RC
13.2	4	mud CG12047-RB
13.3	3	**RpL22**
13.4	3	**RpL22**
14.1	ND	CG14112 (SNCF)
14.2	ND	CG14112 (SNCF)
15.1	4	hth CG17117-RC
